# In vitro antimicrobial activity of natural toxins and animal venoms tested against *Burkholderia pseudomallei*

**DOI:** 10.1186/1471-2334-6-100

**Published:** 2006-06-20

**Authors:** R Perumal Samy, A Pachiappan, P Gopalakrishnakone, Maung M Thwin, Yap E Hian, Vincent TK Chow, Ho Bow, Joseph T Weng

**Affiliations:** 1 Venom and Toxin Research Programme, Department of Anatomy, Yong Loo Lin School of Medicine, National University of Singapore, Singapore - 117597; 2 Department of Microbiology, Yong Loo Lin School of Medicine, National University of Singapore, Singapore - 117597

## Abstract

**Background:**

*Burkholderia pseudomallei *are the causative agent of melioidosis. Increasing resistance of the disease to antibiotics is a severe problem in treatment regime and has led to intensification of the search for new drugs. Antimicrobial peptides are the most ubiquitous in nature as part of the innate immune system and host defense mechanism.

**Methods:**

Here, we investigated a group of venoms (snakes, scorpions and honey bee venoms) for antimicrobial properties against two strains of Gram-negative bacteria *Burkholderia pseudomallei *by using disc-diffusion assay for *in vitro *susceptibility testing. The antibacterial activities of the venoms were compared with that of the isolated L-amino acid oxidase (LAAO) and phospholipase A_2 _(PLA_2_s) enzymes. MICs were determined using broth dilution method. Bacterial growth was assessed by measurement of optical density at the lowest dilutions (MIC 0.25 mg/ml). The cell viability was measured using tetrazolium salts (XTT) based cytotoxic assay.

**Results:**

The studied venoms showed high antimicrobial activity. The venoms of *C. adamanteus, Daboia russelli russelli, A. halys, P. australis, B. candidus *and *P. guttata *were equally as effective as Chloramphenicol and Ceftazidime (30 μg/disc). Among those tested, phospholipase A_2 _enzymes (crotoxin B and daboiatoxin) showed the most potent antibacterial activity against Gram-negative (TES) bacteria. Naturally occurring venom peptides and phospholipase A_2 _proved to possess highly potent antimicrobial activity against *Burkholderia pseudomallei*. The XTT-assay results showed that the cell survival decreased with increasing concentrations (0.05–10 mg/mL) of *Crotalus adamanteus *venom, with no effect on the cell viability evident at 0.5 mg/mL.

**Conclusion:**

This antibacterial profile of snake venoms reported herein will be useful in the search for potential antibacterial agents against drug resistant microorganisms like *B. pseudomallei.*

## Background

Gram-negative *Burkholderia pseudomallei*, the causative agent of melioidosis, are found widely in soil and surface water throughout the tropics. High incidence of melioidosis has been found particularly in Southeast Asia and Northern Australia [1,2]. A number of cases have been reported in Malaysia [3], Thailand [4], Northern Australia [5], South China [6], Taiwan [7], South India, Africa and America. The majority of adult patients develop acute pulmonary or septicaemic illness with high mortality rates [8], or subacute melioidosis, characterized by multiple-abscess formation. In cases of septicaemic melioidosis, which is associated with a vigorous inflammatory cytokine response [9], septic shock continues to be a major cause of morbidity and mortality in patients. Tumor necrosis factor (TNF) is involved in the acquisition of melioidosis, and is also related to disease severity [10].

Antibiotic resistance has been of great concern during the last decades due to the extensive clinical use of classical antibiotics [11]. Currently available antimicrobials fail to lower the mortality rate of melioidosis [12,13]. *B. pseudomallei *demonstrate high levels of resistance to the action of cationic antimicrobial peptides such as polylysine, protamine sulfate, human neutrophil peptides (HNP-1), and polymyxins [14,15]. Therefore, it is of considerable interest to develop antimicrobials with a new mechanism(s) of action which can potentially evade the emergence of drug resistance. In the search for such new agents, we are looking for effective bactericidal peptides from multi-cellular animals that have potential activity against *B. pseudomallei*. The antimicrobial peptides are ubiquitous in nature as part of the innate immune system and host defence mechanisms. They have been increasingly recognized as a critical first line of defence against many pathogens isolated from various sources [16].

Proteinacious components such as neurotoxin, cytotoxin, myotoxin, protease and nuclease are found in varying quantities in snake venoms depending on the species. It has been reported that the basic myotoxic PLA_2 _enzymes, (myotoxin II) of *Bothrops asper *[17] and BnpTx I of *Bothrops neuwiedi pauloensis *[18], possess bactericidal activity against *E. coli *and *S. aureus*. The crude venom and enzymes (LAAO1 and LAAO2) from *Pseudechis australis *are also known to have antibacterial effects against gram (+) and gram (-) bacteria [19]. Besides the strong haemolytic activity shown against sheep erythrocytes, the magainin-type polycationic antimicrobial peptides (pandinin 1 & 2) from the venom of African scorpion *Pandinus imperator *demonstrate high antimicrobial activity against a range of gram (+) and gram (-) bacteria [20]. Moreover, anti-bacterial peptides called the cecropins and defensins have been identified in the hemolymph of scorpions [21]. They are active against bacteria through lysis of their cells thus causing death of the microorganisms. Bactericidal peptides (lycotoxins) have also been identified in the venom of the spider *Lycosa singoriensis *[22], while another family of peptides (cupiennius) with bactericidal activity has been isolated from the venom of a different species of *Cupiennius salei *[23]. Melittin is an important venom component of *Apis mellifera *(common honey bee) which is more active against gram (+) and gram (-) bacteria [24,25]. The highly cationic and salt insensitive cathelicidins of mammalian origin are also reported to have a broad-spectrum of antimicrobial activities [26]. The peptides differ widely in sequence and structure, yet kill bacteria by thinning and disrupting the bacterial membrane [27], and as a result these peptides are important as unique source leads for the development of new therapeutic agents [28]. The above examples thus emphasize the fact that animal toxins or peptides can be rich sources of antimicrobials which may be useful as therapeutics in human.

Although a large number of bioactive components have previously been isolated and characterized from snake venoms, no systematic search for antibacterial components from animal venoms has yet been reported. In our previous studies, majority of the venoms exerted a broad spectrum of antimicrobial activity against various gram-negative and gram-positive bacteria (Journal of Applied Microbiology – under revision). We describe here the antimicrobial properties of 34 different venoms of scorpions, snakes and honey bee, along with those of nine purified venom phospholipase A_2 _enzymes and two L-aminoacid oxidase, evaluated against multi-drug resistant pathogen obtained from the patient with melioidosis (*B. pseudomallei *strains KHW and TES).

## Methods

### Venoms

Lyophilized venoms – *Acanthophis augtra, Acanthophis antarcticus, Acanthophis praelongus, Acanthophis Pyrrhus, Androctonus australis, Bungarus candidus, Hyrophis cyanocinctus, Naja naja naja, Notechis aterater, Naja sumatrana, Naja kaouthia, Pseudechis australis, Pseudechis guttata, Pseudechis porphyriacus, Pseudechis colletti, Pseudonaja inframaggula, Pseudonaja nuchalis, Pseudonaja textilis, Pseudechis affinis, Rhabdophis tigrinus, Oxyuranus scutellatus, Agkistrodon halys, Bitis gabonica rhinoceros, Crotalus adamanteus, Echis carinatus, Daboia russelli russelli, Daboia russelli siamensis, Trimeresurus wagleri, Apis mellifera, Bothotus hottenlota, Buthotus hottenota hottenota, Buthus martensii *and *Naja naja naja *venoms were extracted from long-term captive specimens. Venoms from captive specimens were collected manually by milking. Each sample of freeze-dried venom was stored at 4°C. The solid venoms were obtained from commercial sources (Venom supplies Pte Ltd, Tanunda, South Australia). L-amino acid oxidase purified from the venom of *B. atrox *and *C. adamanteus *were obtained commercially (Sigma Aldrich, St Louis, MO, USA).

### Antibacterial effects of venoms

Each sample of freeze-dried venom was dissolved in 1 mL of 50 mM Tris-HCl buffer (pH 7.4), vortexed (Labnet VX100), and filtered using 0.22 mμ syringe filter (Millipore, NY, USA) before storage at 4°C. All the venoms were tested for their antibacterial activity against gram-negative bacteria *Burkholderia pseudomallei *(KHW) and *Burkholderia pseudomallei *(TES) by disc-diffusion susceptibility tests were performed following standards recommended by the NCCLS [29] with some modifications. Bacterial inoculums (200 μL of a 0.1 A_600 _culture containing 3.2 × 10^8 ^colony forming units cfu/mL) were spread by using a sterile cotton swab onto 20 mL of sterile TS agar plates (90 mm diameter). The surface of the medium was allowed to dry for about 3 min. Sterile paper discs (7 mm diameter) were then placed onto the TS agar surface and 20 μL (0.1 mg/mL) of venom sample was added per disc in 5 replicates. Disc containing 20 μL of Tris-HCl buffer served as a normal control and discs containing antibiotics were used as drug controls. The plates were incubated at 37°C for 24 h, following which the diameter of inhibition zones were measured and calculated, and the resultant activities compared with purified PLA_2 _enzymes.

### Purification of phospholipase A_2 _(PLA_2_) enzymes

Phospholipase A_2 _enzymes were purified from their corresponding crude venoms as described [30] with minor modification. In brief, the venom (0.4 g/4 ml) was fractionated on a Superdex-G75 column with Tris-HCl buffer (50 mM, pH 7.4) as an eluant, followed by further purification on a RP-HPLC Jupiter C18 column (AKTA explorer Workstation, Amersham Pharmacia Biotech, Sweden), eluted with a linear gradient of 80% acetonitrile in 0.1% trifluoroacetic acid. Elution of proteins was monitored at 280 nm and 215 nm. The homogeneity of purified PLA_2_s was determined by using MALDI-TOF on a Voyager DE-STR Biospectrometry workstation (Applied Biosystem CA, USA).

### Antibacterial effects of purified PLA_2_

All the venom enzymes used in the experiment have been purified by successive chromatographic steps with the final purity of at least 95% as assessed by Reversed-phase HPLC. The activities of the following phospholipase A_2 _enzymes purified from the venoms of different snake species (in parenthesis) – crotoxin A (*Crotalus durissus terrificus*), crotoxin B (*Crotalus durissus terrificus*), ammodytoxin A (*Vipera ammodytes ammodytes*), mojavetoxin (*Crotalus scutulatus scutulatus*), β-bungarotoxin (*Bungarus multicinctus*), taipoxin (*Oxyuranus scutellatus scutellatus*), mulgatoxin (*Pseudechis australis*), *Daboiatoxin *(*Daboia russelli russelli*), honey bee (*Apis mellifera*) venom phospholipase A_2 _– and two L-amino acid oxidase – LAAO (*B. atrox*), LAAO (*C. adamanteus*) were evaluated. 0.5 μmoles of each of the enzyme dissolved in 500 μL of 50 mM Tris-HCl (pH7.4) buffer was examined. *In vitro *antimicrobial activity was determined by the previously described disc-diffusion method [29] with some modifications.

### MIC determinations

In preliminary experiments, the disc-diffusion assay for determining antibacterial effects of crude venoms was compared to the activity of isolated phospholipase A_2 _(PLA_2_) enzymes using broth-microdilution method. Differences in data between these two assays were observed. Thus, we used the broth-microdilution technique to test the activities of the phospholipase A_2 _against pathogen from patients by comparing their activities to the activity of the antibiotics. Bacteria from frozen suspensions were sub-cultured onto Tryptic soya (TS) agar plates and passaged twice prior to susceptibility testing. The bacteria were grown in Tryptic soy broth (TSB) for 5 – 7 h (exponential phase) before adjusting their concentration to a 0.5 McFarland turbidity standard. The adjusted bacterial cultures were diluted to approximately (A_600 _of 0.8) 3.2 × 10^8 ^colony forming units (cfu/mL). The enzymes (PLA_2S_) to be examined were dissolved in 1 M Tris-HCl buffer (pH 7.4) for determination of the activities (MIC). The bacteria were washed and incubated with the enzymes in appropriate buffer. MICs were determined by the broth microdilution method recommended by the NCCLS [31,32], for which serial dilutions of each enzyme solutions were prepared (final concentration 0.5, 0.25, 0.125, 0.0625 and 0.03125 mg/mL) in microtiter trays with appropriate medium (TSB). Each dilution series included control wells containing bacteria without enzymes. A total of 200 μL of the adjusted inoculum of 10^5 ^cfu/mL was added to each well (96-well plates). The culture trays were incubated at 37°C for 24 h with shaking at 230 rpm. The inhibition of bacterial growth was determined by measuring the absorbance at 560 nm (Molecular Devices E precision microplate reader). The MIC was taken as the lowest concentration of phospholipase A_2 _enzyme that inhibited visible growth of the organism. The results given are mean values of three independent determinations.

### Biochemical characterization

#### Phospholipase A_2 _enzyme activity

Phospholipase A_2 _(PLA_2_) catalyzes the hydrolysis of phospholipids at the *sn*-2 position yielding a free fatty acid and a lysophospholipid. The Cayman Chemical secretory PLA_2 _(sPLA_2_) assay kit was used for the measurement of enzyme activity. This assay uses the 1, 2-dithio analog of diheptanoyl phosphatidylcholine which serves as a substrate for most PLA_2_s [33]. sPLA_2 _specific activity is expressed as μmole/min/mg protein.

#### Protein assay

The protein concentration of the crude venom solutions was determined using the Bio-Rad protein assay reagent [34] and bovine serum albumin as a standard.

#### Cell Proliferation assay (XTT-based cytotoxicity assay)

The human macrophage cell line (U-937) was purchased from ATCC (Virginia, USA) and cell Proliferation Kit II was from Roche Applied Sciences (Singapore). Sterile Roswell Park Memorial Institute (RPMI) medium, Fetal Bovine Serum (FBS), 1 M Tris-HCl buffer (pH 7.4), and 10 mM HEPES were purchased from National University Medical Institute (NUMI), Singapore. All chemicals were of analytical and cell culture grade. Human macrophage (U-937) cell line was cultured in 72 cm^2 ^flasks at a density of 1 × 10^7^cells/12 mL in RPMI culture medium supplemented with 10% fetal bovine serum (FBS), and 1 ml of HEPES. The cell viability was measured using tetrazolium salts (XTT) as described [35]. Briefly, the cells were allowed to adhere to the bottom of the flask for overnight at 37°C in a humidified atmosphere of 5% CO_2 _and 95% air. The culture medium was changed three times a week. To analyze the initial events of venom-mediated cell viability, five different venoms were applied to cultured macrophage cell lines with each venom tested at different concentrations (0.05 – 10 mg/mL) and varied time intervals (12, 24, and 48 h). Cell proliferation was spectrophotometrically quantified using an ELISA plate reader at 490 nm. All assays were prepared in triplicates and repeated thrice. The cytotoxicity of purified PLA_2 _enzymes was also tested at different concentrations (0.05–10 μg/mL) using XTT assay.

### Statistical analysis

The results (mean ± S.D, n = 5) were analysed by one way-ANOVA with repeated measures to analyze factors influencing the zone size of growth inhibition, and for comparison with standard drugs.

## Results and discussion

**Figure 1:**
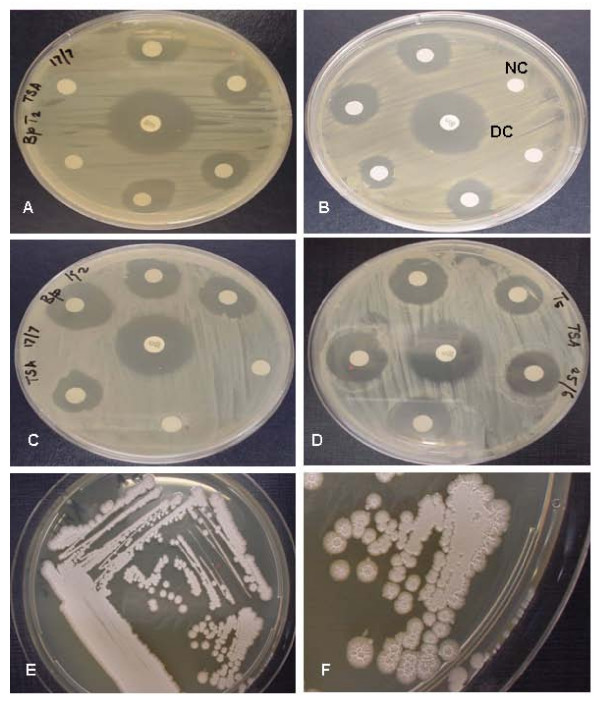
Antimicrobial activities of crude venoms (each disc contained 20 μl of 100 μg/ml) of different snake species tested against gram-negative *Burkholderia pseudomallei*. Following 24 h incubation at 37°C, zone of inhibition given by each venom was compared with that of the standard drug chloramphenicol (30 μg/disc). Inhibition zones against *Burkholderia pseudomallei *given by venoms of: (A) *Daboia russelli russelli*, (B) *Agkistrodon halys*, (C) *Crotalus adamanteus*, (D) *Bitis gabonicarhinoceros*. Rough wrinkled morphological features of gram-negative *Burkholderia pseudomallei *bacteria were grown on Tryptic Soy agar plates at 37°C. (E) bacilli after 36 h incubation, (F) prominent bacilli after 72 h incubation.

In the present study, both the strains of *B. pseudomallei *(KHW & TES) proved to be highly or intermediately susceptible to various snake venoms at the tested concentration (Table 1). The venom from crotalid (*C. adamanteus*) species showed the most potent antibacterial activity and exhibited larger zone of inhibition on *B. pseudomallei *(Fig. 1) than the venoms of a viperid, *D. russelli russelli *and an Australian elapid, *Pseudechis australis*. Crotalid venoms have previously been reported to have broad activity against aerobic gram-positive and negative bacteria [36], but this is the first report showing the potent antibacterial property of *C. adamanteus *venom against the drug resistant *B. pseudomallei*. The antibacterial effects of crotalid, viperid and elapid venoms established in the present study may likely be due to the cyto-toxins (direct lytic factors) and phospholipase A_2 _enzymes contained in those venoms [37]. Association between L-amino acid oxidase activity (LAAO1 and LAAO2) and antibacterial property of *Pseudechis australis *venoms has previously been suggested [19]. However, according to our results, the 5 different venoms of *C. adamanteus, Daboia russelli russelli*, *P. australis, P. guttata *and *A. halys *exhibited stronger antimicrobial activity against both strains of *B. pseudomallei *than that shown by the L-amino acid oxidase enzymes of *C. adamanteus *and *B. atrox *venoms, thus suggesting that LAAO activity alone may not be solely responsible for the antibacterial activity of these venoms.

**Table 1:** Antibacterial properties of animal venoms tested against *Burkholderia pseudomallei *at 0.1 mg/mL concentration.

Common name	Scientific name	Phospholipase A_2 _enzymatic activity		Yield of protein (mg/mL)	Micro-organisms	
				
		Total activity (μmoles/min)	Specific activity (μmoles/min/mg protein)		*B. pseudomallei *(KHW)	*B. pseudomallei *(TES)
**Elapidae**						
Death adder	*Acanthophis augtra*	3.74	74.5 ± 0.5	0.1	-	-
Common death adder	*Acanthophis antarcticus*	190	487.5 ± 0.96	0.78	16.6 ± 0.43	14.3 ± 0.34
Northern death adder	*Acanthophis praelongus*	241.2	1416 ± 3.84	0.34	8.90 ± 0.23	8.47 ± 0.21
Desert death adder	*Acanthophis pyrrhus*	138	1150 ± 0.98	0.24	14.7 ± 0.23	16.5 ± 0.32
Hector	*Androctonus australis*	22.2	85.2 ± 0.27	0.52	-	8.13 ± 0.14
Malayan krait	*Bungarus candidus*	34.9	166.3 ± 0.56	0.42	-	-
Sea snake	*Hydrophis cyanocinctus*	3.3	40.5 ± 0.56	0.16	-	-
Indian cobra	*Naja naja naja*	293.4	1333 ± 1.7	0.44	12.2 ± 0.16	10.2 ± 0.09
Kreft's tiger snake	*Notechis aterater*	41.96	183.1 ± 0.59	0.46	-	-
Spitting cobra	*Naja sumatrana*	406.5	903.5 ± 0.6	0.90	-	-
Cobra	*Naja kaouthia*	228.4	1904 ± 3.8	0.24	8.17 ± 0.15	-
King brown snake	*Pseudechis australis*	434.5	3949 ± 3.2	0.22	27.7 ± 0.13	29.8 ± 0.105
Speckled brown snake	*Pseudechis guttata*	308.5	791 ± 2.0	0.78	25.4 ± 0.19	26.8 ± 0.109
Red-bellied black snake	*Pseudechis porphyriacus*	726.7	3303 ± 1.7	0.44	16.8 ± 0.15	14.2 ± 0.17
Collett's snake	*Pseudechis colletti*	15.7	111.8 ± 0.89	0.28	-	-
-	*Pseudonaja inframaggula*	1995	5945 ± 26.6	0.66	7.7 ± 0.16	8.32 ± 0.11
Western brown snake	*Pseudonaja nuchalis*	162.8	361.8 ± 0.6	0.90	8.2 ± 0.08	9.30 ± 0.11
Eastern brown snake	*Pseudonaja textilis*	416.8	832.3 ± 0.6	1	14.2 ± 0.09	8.10 ± 0.14
Dugite	*Pseudechis affinis*	218.7	376.8 ± 0.9	1.16	-	-
Tiger keelback	*Rhabdophis tigrinus*	36.3	259.1 ± 0.42	0.28	-	-
The coastal taipan	*Oxyuranus scutellatus*	1275.7	6075 ± 0.98	0.63	-	-
**Viperidae**						
Pallas	*Agkistrodon halys*	86.5	157.4 ± 0.20	1.1	20.4 ± 0.14	26.4 ± 0.08
Puff adder	*Bitis arietans*	124.1	248.2 ± 0.27	0.54	18.2 ± 0.16	16.2 ± 0.17
West African gaboon viper	*Bitis gabonica rhinoceros*	126.5	452.4 ± 0.57	0.56	16.0 ± 0.19	14.5 ± 0.26
Common lancehead snake	*Bothrops atrox *(L-amino acid oxidase)	3.8	6.36 ± 0.06	1.2	24.4 ± 0.19	26.2 ± 0.19
South American rattlesnake	*Crotalus adamanteus *(L-amino acid oxidase)	34.2	201.3 ± 0.21	0.34	25.4 ± 0.208	23.4 ± 0.18
South American rattlesnake	*Crotalus adamanteus*	236.4	619.4 ± 0.46	1.56	30.0 ± 0.23	28.3 ± 0.23
Saw-scaled viper	*Echis carinatus*	53.4	106.5 ± 0.49	1.4	8.0 ± 0.14	7.5 ± 0.15
Indian Russell's viper	*Daboia russelli russelli*	392.8	785.2 ± 0.40	1.36	29.9 ± 0.12	28.6 ± 0.16
Burmese Russell's viper	*Daboia russelli siamensis*	262.4	524.4 ± 0.44	1.24	16.2 ± 0.15	15.6 ± 0.18
Wagler's pit viper	*Trimeresurus wagleri*	4.6	38.2 ± 0.26	0.24	15.1 ± 0.14	16.3 ± 0.14
**Apiidae**						
Honeybee venom	*Apis mellifera*	3.7	20.5 ± 0.1	0.36	7.22 ± 0.23	12.3 ± 0.21
**Scorpionidae**						
Black scorpion	*Androctonus crasicuda*	3.6	69.5 ± 0.3	0.104	-	-
Scorpion	*Buthotus hottenlota*	4.4	10.4 ± 0.2	0.86	-	-
Scorpion	*Buthotus hottenota hottenota*	5.1	38.8 ± 0.1	0.26	-	-
Chinese red scorpion	*Buthus martensii *Karsch.	4.6	90.4 ± 0.2	0.102	-	-
Ceftazidime (CF)	30 μg/disc	-	-	-	28.9 ± 0.56	31.7 ± 0.78
Chloramphenicol (C)	30 μg/disc	-	-	-	28.5 ± 0.37	30.0 ± 0.12
Streptomycin (S)	10 μg/disc	-	-	-	27.3 ± 0.25	26.4 ± 0.29

Values of Phospholipase A_2 _enzymatic activity (μmoles/min/mg protein) are presented as mean ± S.D. (n = 10) of ten replicates.

*The values given as mean ± S.D. (n = 5) represent a venom inhibition zone in mm, including the 7 mm diameter of the disc, after 24 h incubation. The bacterial inoculum per plate contained 3.2 × 10^8 ^colony forming units (cfu/mL) which were spread onto the TS agar surface with sterile cotton swap. Sterile paper discs (7 mm diameter) were placed onto the TS agar surface and 20 μL of venom solution (0.1 mg/mL) added. Micro-organisms: *B.p (KHW)-Burkholderia pseudomallei *(KHW) and *B.p (TES) Burkholderia pseudomallei *(TES), Control (0); No activity (-), Drug controls: Chloramphenicol (30 μg/disc); Ceftazidime (30 μg/disc); Streptomycin (10 μg/disc).

In the present study, we have therefore tested a variety of phospholipase A_2 _enzymes purified from different snake venoms against *Burkholderia pseudomallei*, and have found that crotoxin B and daboiatoxin display the strongest antibacterial activity against both the strains of *B. pseudomallei *(KHW & TES) (Table 2). Crotoxin B is a basic neurotoxic phospholipase A_2 _(*Crotalus durissus terrificus*) containing three chain protein that enhances the lethal potency of crotoxin [38], while daboiatoxin is a monomeric PLA_2 _(*Daboia russelli siamensis*) with strong neurotoxic and myotoxic activities [30]. Moreover, mulgatoxin (myotoxic PLA_2 _of *P. australis*) and bee (*Apis mellifera*) venom PLA_2 _have also been found to exert significant antibacterial activity against both strains (KHW & TES) of *B. pseudomallei*.

**Table 2:** *In vitro *antibacterial activity of purified phospholipase A_2 _enzymes from snake venoms.

Phospholipase A_2_ enzymes (PLA_2_s)	Snake species (Scientific name)	Mol. wt.(kDa)	Conc. (mg/mL)	Micro-organisms	
				
				*B. pseudomallei *(Strain KHW)	*B. pseudomallei *(Strain TES)
Crotoxin A (CA)	*Crotalus durissus terrificus*	23.5	0.5 mg/mL	-	-
Crotoxin B (CB)	*Crotalus durissus terrificus*	23.5	0.5 mg/mL	24.8 ± 0.089	27.6 ± 0.133
Ammodytoxin A	*Vipera ammodytes ammodytes*	13.8	0.5 mg/mL	-	-
Mojave toxin	*Crotalus scutulatus scutulatus*	23.5	0.5 mg/mL	-	-
β-Bungarotoxin	*Bungarus multicinctus*	20.5	0.5 mg/mL	-	-
Taipoxin	*Oxyuranus scutellatus scutellatus*	45.6	0.5 mg/mL	-	
Mulgatoxin	*Pseudechis australis*	13.2	0.5 mg/mL	20.5 ± 0.075	22.7 ± 0.117
Daboiatoxin (DbTx)	*Daboia russelli siamensis*	15.0	0.5 mg/mL	24.8 ± 0.103	26.2 ± 0.121
Bee venom PLA_2_	*Apis mellifera*	19.0	0.5 μg/mL	18.3 ± 0.089	20.4 ± 0.075

*The values given represent a venom inhibition zone in mm, including the 7 mm diameter of the disc, after 24 h incubation. The bacterial inoculum per plate contained 3.2 × 10^8 ^cfu/mL forming units which were spread onto the TS agar surface with sterile cotton swap. Sterile paper discs (7 mm diameter) were placed onto the TS agar surface and 20 μL of enzymes (0.5 mg/ml concentration) added, Control (0); No activity (-).

**Figure 2:**
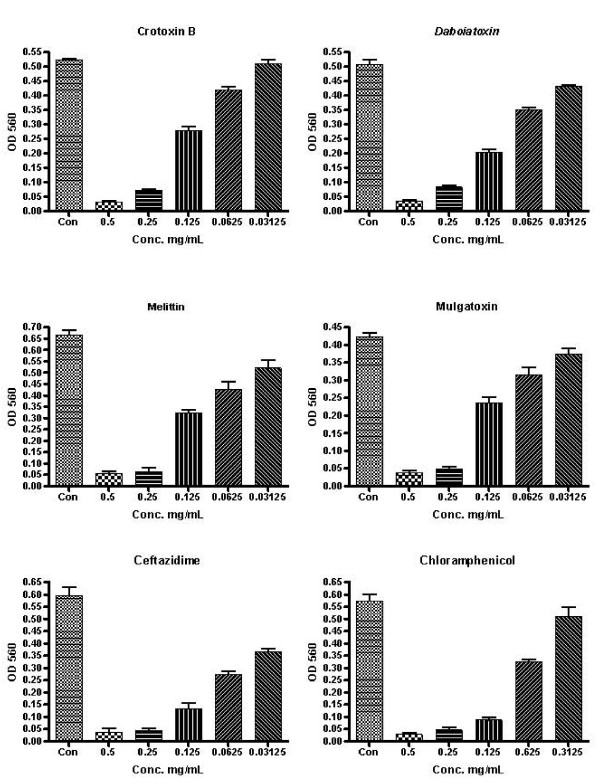
Phospholipase A_2 _activity against pathogen from patients with KHW. Micro-dilution technique was used to test the MICs of PLA_2_s as compared to that of the antibiotics. The values are the optical density read at 560 nm (means ± S.D.) from a single experiment performed in triplicates.

**Figure 3:**
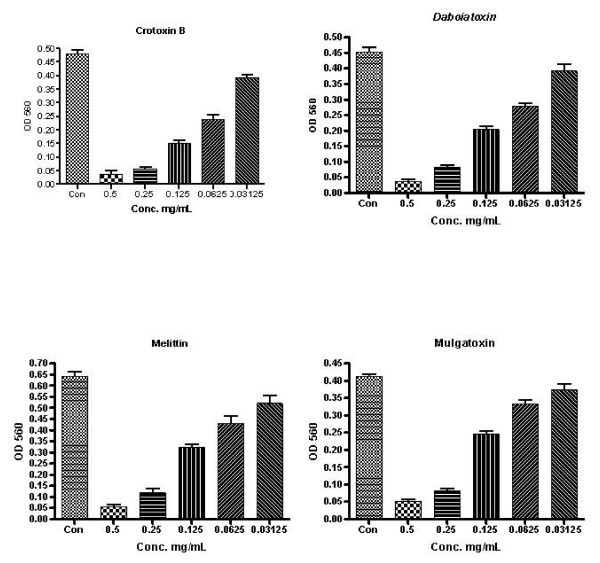
Phospholipase A_2_activity against pathogen from patients with TES. Micro-dilution technique was used to test the MICs of PLA_2_s as compared to that of the antibiotics. The values are the optical density read at 560 nm (means ± S.D.) from a single experiment performed in triplicates.

The PLA_2 _enzymes showing potent inhibitory activity were further studied for minimum inhibitory concentrations (MICs) (Fig. 2, 3). Among those examined, the two purified PLA_2 _enzymes (crotoxin B and daboiatoxin) showed stronger inhibitory activity at a lower dilution (MICs 0.25 mg/mL) against *B. pseudomallei *(TES) than melittin and mulgatoxin. The two isolates (TES and KHW) of *B. pseudomallei *showed various levels of sensitivity to the two PLA_2 _toxins with MICs ranging between 0.5 and 0.03125 mg/ml. The susceptibility of crotoxin B (69%) and daboiatoxin (63%) against TES was more or less equal to that of chloramphenicol and ceftazidime against TES (Table 3). The antibiotics chloramphenicol (70%) and ceftazidime (80%) were highly susceptible against *B. pseudomallei *TES & KHW, but MIC breaking points of antibiotics were recorded at the lowest dilution (MICs 0.125 mg/mL) than the PLA_2 _toxins. Our results corroborate with the previous report on the conventional four-drug regimen which has been replaced by ceftazidime for acute treatment. However, the chlroarmphenicol has also been given for the first 8 weeks of oral treatment [39]. In the present study, the antibiotic activity was gradually declined at the dilution 0.03125 mg/mL against *B. pseudomallei *(KHW) and (TES). The rate of resistance of ceftazidime and chloramphenicol against KHW was 20% and 30%, respectively. In a randomised trial previously reported, 161 severe meloidosis patients were treated with ceftazidime (120 mg/kg) + chloramphenicol (100 mg/kg) + doxycycline (4 mg/kg) + TMP/SMX for less than 7 days. The mortality was 37% for ceftazidime and 74% for chloramphenicol [40]. However, in our study, melittin was slightly more active than mulgatoxin against *B. pseudomallei *(strain KHW) at MIC 0.25 mg/mL. It showed very weak MICs (Fig. 2, 3) when compared with chloramphenicol and ceftazidime. Other PLA_2_s (ammodytoxin A, Mojave toxin, β-bungarotoxin and taipoxin) however, lacked any activity against both isolates of *B. pseudomallei *at all tested dilutions (0.5 – 0.03125 mg/mL). Based on previous studies using cationic antibacterial peptides, cathelicidin-derived peptides had modest MIC against *S. maltophilia *and *A. xylosoxidans *(1.0 to > 32 mg/mL), but none inhibited *Burkholderia cepacia *[41]. Moreover, another cationic peptide (hBD-3) that was proven highly or intermediately effective (MBCs >100 μg/mL) against 23 tested strains did not show any effect against *Burkholderia cepacia *at 50 μg/mL [42]. When the MIC of peptide D2A21 was compared with that of the tracheal antimicrobial peptide (TAP), the former peptide displayed greater potency than TAP against *P. aeruginosa *at 0.125 – 4 mg/mL, *S. aureus *at 0.25 – 4 mg/mL, and *Burkholderia cepacia *at 32 to >64 mg/mL, respectively [43]. Relative to the MIC of these cationic antimicrobial peptides, crotoxin B appears to have a much lower dose (MIC 0.25 mg/mL) against *B. pseudomallei*. MIC value of PLA_2_s are more or less equally comparable to that of ceftazidime (MICs 0.125 mg/mL), a drug of choice for melioidosis infections in humans. The rat model of lung infection (14 days) showed that the *B. cenocepacia *LPS inner core oligosaccharide is needed for resistance to three structurally unrelated antimicrobial peptides for in vivo survival in a rat model of chronic lung infection [44].

**Table 3:** MIC breakpoints for ceftazidime and chloramphenicol when compared to that of purified PLA_2_s enzymes.

Phospholipase A_2_ enzymes (PLA_2_s)	MIC mg/mL	*B. pseudomallei *(strain KHW)					MIC mg/mL	*B. pseudomallei *(strain TES)				
	
	Ctrl	0.5	0.25	0.125	0.0625	0.03125	Ctrl	0.5	0.25*	0.125^a^	0.0625	0.03125
Crotoxin B (CB)	0.65	0.012 (64%)	0.04 (61%)	0.33 (32%)	0.43 (32%)	0.56 (9%)	0.73	0.05 (73%)	0.06 (69%)	0.33 (40%)	0.43 (30%)	0.52 (21%)
Daboiatoxin (DbTx)	0.65	0.26 (39%)	0.09 (56%)	0.37 (28%)	0.49 (16%)	0.55 (10%)	0.68	0.03 (65%)	0.05 (63%)	0.24 (20%)	0.36 (8%)	0.43 (1%)
Bee venom PLA_2_	0.48	0.038 (38%)	0.056 (42%)	0.15 (33%)	0.24 (24%)	0.39 (9%)	0.44	0.03 (45%)	0.06 (38%)	0.27 (17%)	0.36 (8%)	0.41 (3%)
Mulgatoxin	0.46	0.07 (39%)	0.083 (37%)	0.21 (25%)	0.28 (18%)	0.39 (7%)	0.43	0.04 (40%)	0.07 (37%)	0.26 (18%)	0.32 (12%)	0.37 (7%)
Chloramphenicol	0.67	0.01 (68%)	0.03 (64%)	0.07 (60%)	0.42 (10%)	0.51 (1%)	0.77	0.026 (74%)	0.04 (73%)	0.07 (70%)	0.34 (41%)	0.37 (38%)
Ceftazidime	0.76	0.08 (58%)	0.05 (61%)	0.04 (62%)	0.24 (24%)	0.44 (22%)	0.89	0.01 (88%)	0.03 (86%)	0.09 (80%)	0.35 (47%)	0.49 (33%)

* MIC values are given as mean of five replicates (n = 5), the bacterial inoculums per plate contained 3.2 × 10^8 ^cfu/mL forming units/well, Control (bacterial inoculums); ^a^The ceftazidime breaking points (MICs 0.125 mg/mL) against TES, *The PLA_2 _toxin breaking points (MICs 0.250 mg/mL) against both the strains of *B. pseudomallei *after 24 h incubation.

When PLA_2 _activity was examined, the highest activity was found in the Australian elapid venoms (*Oxyuranus scutellatus>Pseudonaja inframaggula*) followed by *Pseudechis australis, Pseudechis porphyriacus, Naja kaouthia, Naja naja naja, Acanthophis praelongus *and *Acanthophis pyrrhus*respectively. In contrast, the remaining venoms of the Apiidae and Scorpionidae showed relatively less phospholipase A_2 _activity than the viperidae venoms (Table 1).

**Figure 4:**
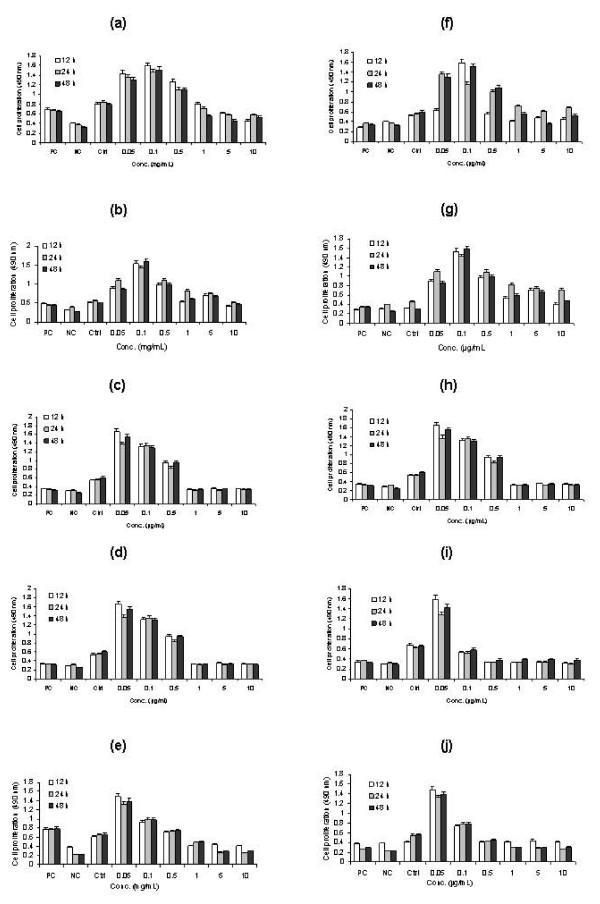
Cell proliferation (U-937 Human macrophage cell line) was determined by XTT assay to evaluate the cytotoxic effect of different venoms (a – e) and PLA_2_s (f – j) exposed at different time intervals. (a) South American rattlesnake (*Crotalus adamanteus*), (b) Russells viper (*Daboia russelli russelli*), (c) King brown (*Pseudechis australis*), (d) Pallas (*Agkistrodon halys*) (e) Speckled brown (*Pseudechis guttata*) (f) Crotoxin B (*Crotalus durissus terrificus*), (g) Daboiatoxin (*Daboia russelli russelli*), (h) Melittin (*Apis mellifera*), (i) Mulgatoxin (*Pseudechis australis*) (j) Crotoxin A (*Crotalus durissus terrificus*). Macrophages were incubated with varying concentrations of venoms (0.05 – 10 mg/ml) and PLA_2_s (0.05 – 10 μg/ml).

**Figure 5:**
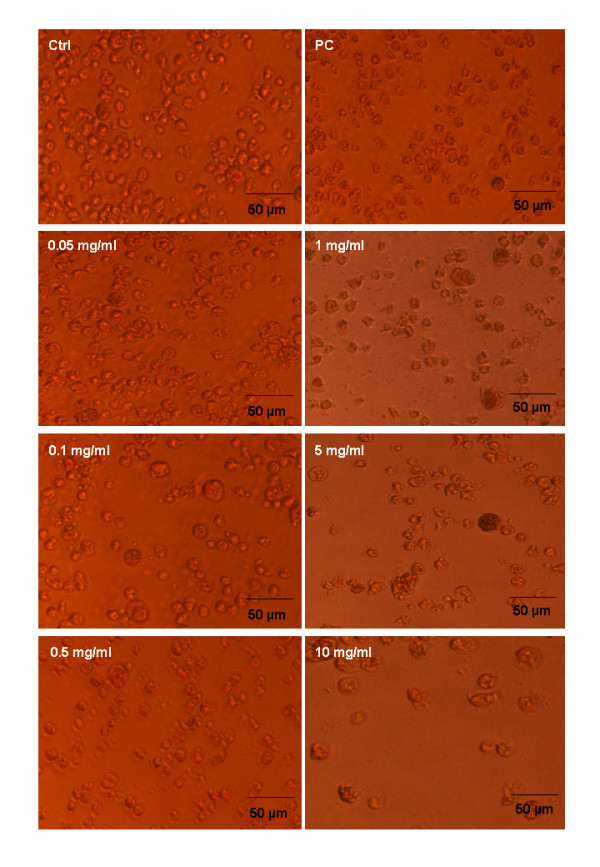
Morphological changes of U-937 Human macrophage cell line after exposure to *Crotalus adamanteus *venom at different concentrations. (Ctrl) macrophage supplemented with medium without any treatment served as control; (PC), cells exposed with ceftazidime as a positive control. Macrophages were incubated with venom at different (0.05–10 mg/mL) concentrations (Other photos not shown).

**Figure 6:**
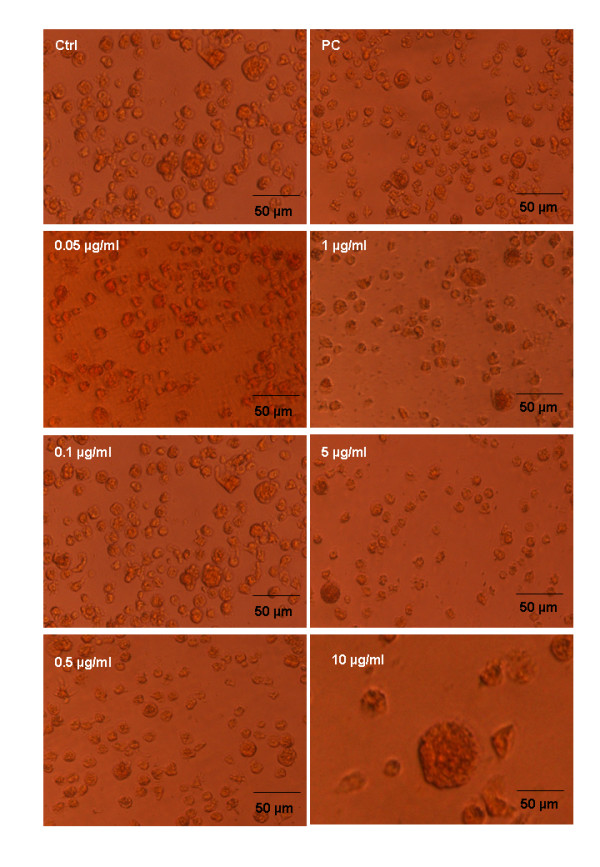
Morphological changes of U-937 Human macrophage cell line after exposure to PLA_2_s at varying concentrations (0.05–10 μg/mL). (Ctrl), control macrophages supplemented with medium without any treatment; (PC), cells exposed with ceftazidime as a positive control.

The survival bar resulting from the XTT assay shows that all five venoms (*C. adamanteus, B. gabonica, P. australis, D. russelli russelli *and *A. halsy *)do not have cytotoxic effects on the proliferation of cells up to 0.5 mg/mL concentration (Fig. 4a–e). However, the higher concentrations (1, 5 and 10 mg/mL) showed severe morphological changes of the cell lines such as membrane disruption and significant cell lyses when compared with control and the positive control (Fig. 5). In contrast, the purified PLA_2_s, crotoxin B and daboiatoxin, did not change the viability (Fig. 4f–g) of cells up to 0.05 – 10 μg/mL concentrations as compared to the control. The cell viability and morphology of macrophage cells were shown (Fig. 6) after exposure to crotoxin B in a dose- and time-dependent manner.

**Figure 7:**
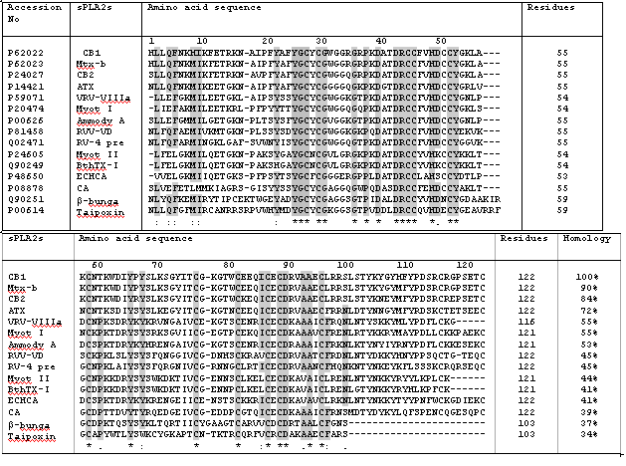
Comparison of the amino acid sequences of Crotoxin basic chain 1, CB1 [*Crotalus durissus terrificus*] phospholipase A_2 _enzymes (Phosphatidylcholine 2-acylhydrolase) with other PLA_2s _of Mojave toxin basic chain, Mtx-b [*Crotalus scutulatus scutulatus*], Crotoxin basic chain 2, CB2 [*Crotalus durissus terrificus*], Agkistrotoxin, ATX [*Agkistrodon halys*], VRV-PL-VIIIa [*Daboia russelli pulchella*], BOTAS (Myotoxin I) *Bothrops asper *(Terciopelo), ATXA, ammodytoxin A precursor [*Vipera ammodytes ammodytes*]; RVV-VD [*Daboia russelli russelli*]; RV-4 precursor [*Daboia russelli siamensis*], BOTAS (Myotoxin II) [*Bothrops asper*], BOTJR (BthTX-I) [*Bothrops jararacussu*], ECHCA (Ecarpholin S) [*Echis carinatus *(Saw-scaled viper)], Crotoxin acid chain precursor (CA) [*Crotalus durissus terrificus*], β-bungarotoxin A6 chain precursor [*Bungarus multicinctus *(Many-banded krait)] and OXYSC taipoxin alpha chain [*Oxyuranus scutellatus scutellatus*] and completely conserved residues in all sequences are bolded and marked by asterisks. The gaps are inserted in the sequences in order to attain maximum homology.

**Figure 8:**
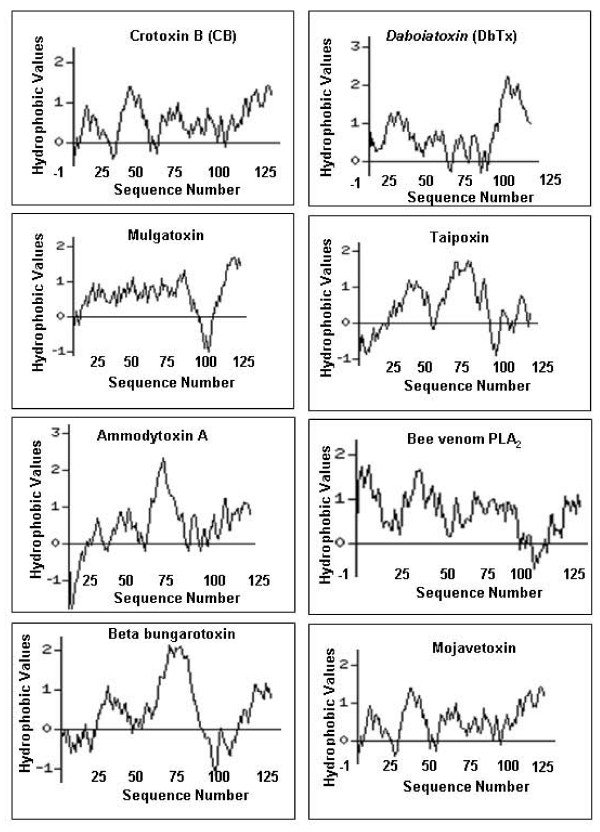
Hydropathic profiles of phospholipase A_2 _enzyme such as crotoxin b, daboiatoxin, mulgatoxin, taipoxin, ammodytoxin A, bee venom PLA_2_, beta-bungarotoxin and mojavetoxin were calculated by using the kyte-doolittle method.

A comparison of the N-terminal sequences of crotoxin B with other snake venom PLA_2_s shows (Fig. 7) that most amino acid residues are conserved in Ca^+2 ^binding and catalytic network regions. The C-terminal segment of crotoxin B, on the other hand, shows a modest difference in the amino acid residues as compared with the C-terminal sequences of other venom PLA_2_s. Moreover, comparison of the hydropathic profiles of different snake venom PLA_2_s reveals that the C-terminal segment of crotoxin B is relatively more hydrophobic than those of other PLA_2_s (Fig. 8). This cationic hydrophobic nature of crotoxin B phospholipase A_2 _enzyme may most likely be responsible for the strong antimicrobial action seen on *B. pseudomallei*.

## Conclusion

The present study indicates that the purified PLA_2 _enzymes (crotoxin B, daboiatoxin) possess strong antibacterial activity against *B. pseudomallei*. The fact that viperidae (*Crotalus adamanteus, Daboia russelli russelli*) and elapidae (*Pseudechis australis*) venoms display more potency than other venoms may be due to the PLA_2 _enzymes present. However, this antibacterial profile of snake venoms reported herein will be useful in the search for potential antibacterial agents against drug resistant microorganisms like *B. pseudomallei*.

## Abbreviations

LAAO – L-amino acid oxidase

PLA_2_s – Phospholipase A_2 _enzymes

MICs – Minimum Inhibitory Concentrations

XTT – Tetrazolium salts

KHW, TES – different isolates of *Burkholderia pseudomallei*

RPMI – Roswell Park Memorial Institute (RPMI) medium

FBS – Fetal Bovine Serum

## Competing interests

The author(s) declare that they have no competing interests.

## Authors' contributions

RPS – Study design, performance of the entire parameters, data analysis and manuscript drafting.

AP – Cell proliferation study using XTT based cytotoxic assay.

PG – Supervision, laboratory support, critical analysis and manuscript edition.

MMT – Manuscript evaluation and critical analysis.

YHH, VTKC, HB – Co supervision of the antimicrobial susceptibility testing, provision of bacterial cultures and laboratory support.

JTW – Assisted in technical support for antimicrobial testing and culturing of bacteria.

## Pre-publication history

The pre-publication history for this paper can be accessed here:


